# Uncommon Extradural Spinal Fibrolipoma in a Child: A Case Report

**DOI:** 10.7759/cureus.77525

**Published:** 2025-01-16

**Authors:** Glennie Ntsambi, Israël Maoneo, Renault Kambere, Larrey Kasereka Kamabu, Antoine Beltchika

**Affiliations:** 1 Neurosurgery, University of Kinshasa, Kinshasa, COD; 2 Neurosurgery, University of Kisangani, Kisangani, COD; 3 Pathological Anatomy, University of Kinshasa, Kinshasa, COD; 4 Neurosurgery, Catholic Unversity of Graben, Butembo, COD; 5 Neurological Surgery, New Deal SARL Hospital/CIMAK, Goma, COD; 6 Neurological Surgery, Makerere University, Kampala, UGA

**Keywords:** epidural spinal cord compression, extradural inflammatory tumor, fibrolipoma, pediatric spinal tumors, surgical excision of tumor

## Abstract

Pediatric spinal tumors include a variety of developmental lesions and uncommon neoplasms that differ significantly from those seen in adults. These conditions are underreported in the sub-Saharan medical literature. We present the case of a 10-year-old girl brought by her family to the University Teaching Hospital of Kinshasa in the Democratic Republic of Congo with progressive lower limb functional impairment.

On admission, the patient was alert, with normal vital signs, pink palpebral conjunctiva, anicteric sclerae, unremarkable cardiopulmonary and spinal examinations, and no spinal deformities. However, palpation revealed tenderness along the dorsal spinous processes from T1 to T10, and a sensory level corresponding to the T4 dermatome was noted. The patient was paraplegic and wheelchair-bound, with a lower limb American Spinal Injury Association (ASIA) motor score of 20/50.

A CT scan of the thoracolumbar spine revealed no disc or vertebral abnormalities. An MRI demonstrated an extensive extradural, intracanal mass from T1 to T10 causing spinal cord compression. The mass exhibited signal characteristics suggestive of a fat-containing lesion.

A posterior surgical approach was performed for tumor excision 20 days after admission. Histopathological analysis confirmed an inflammatory fibrolipomatous tumor. Postoperatively, the patient showed significant neurological improvement after three months of physiotherapy. This report highlights the diagnostic and therapeutic challenges associated with rare spinal lesions in children.

## Introduction

Spinal tumors are significantly rarer than intracranial neoplasms, with an annual incidence of approximately one case per million children [1,2]. They account for 1% to 10% of all central nervous system tumors in the pediatric population [3]. Spinal tumors can be classified as intramedullary or extramedullary. Extramedullary lesions are further subdivided into intradural and extradural tumors. Among children, astrocytomas are the most common intramedullary tumors [4].

Extradural tumors encompass lesions located in the epidural, paraspinal, or vertebral regions. These include osteoid osteomas, osteoblastomas, giant cell tumors, aneurysmal bone cysts, vertebral hemangiomas, fibrous dysplasias, eosinophilic granulomas, schwannomas, embryonic tumors (epidermoid, dermoid, teratoma, lipoma), parasitic cysts, and histiocytosis. Malignant lesions include sarcomas, neuroblastomas, ganglioneuroblastomas, primitive neuroectodermal tumors (PNET), and lymphomas [4-6].

Despite similarities in symptomatology between adults and children, diagnosing spinal tumors in pediatric patients is challenging due to their heterogeneous presentation and the difficulty in conducting thorough neurological examinations in young patients. Consequently, delays in diagnosis are common, often leading to the progression of spinal cord compression syndromes. These syndromes manifest as spinal, radicular, or sublesional symptoms [7].

An MRI is the preferred diagnostic modality, offering a superior resolution for identifying lesion characteristics, localization, and relationships with adjacent neural structures, supporting surgical planning [8]. This case report describes an unusual pediatric extradural inflammatory fibrolipoma; its clinical, diagnostic, therapeutic, and post-treatment features; and the challenges encountered.

## Case presentation

The patient, a 10-year-old girl from the Republic of Angola, a southern neighboring country of the Democratic Republic of Congo (DRC), presented with progressive functional impairment of the lower limbs. Her symptoms began five months prior with lumbar pain, for which she was treated with analgesics and anti-inflammatory neuromodulators at a peripheral hospital near Kinshasa, DRC, without relief. Subsequently, she developed bilateral lower limb pain and paralysis, prompting her transfer to the University Teaching Hospital of Kinshasa.

Upon admission, the patient complained of back pain and functional impairment of the lower limbs. She was alert, hemodynamically stable, and exhibited no spinal deformities. Examination revealed tenderness along the T1-T10 spinous processes and a sensory level at the T4 dermatome. Lower limb examination demonstrated paraplegia, with an American Spinal Injury Association (ASIA) motor score of 20/50.

**Figure 1:**
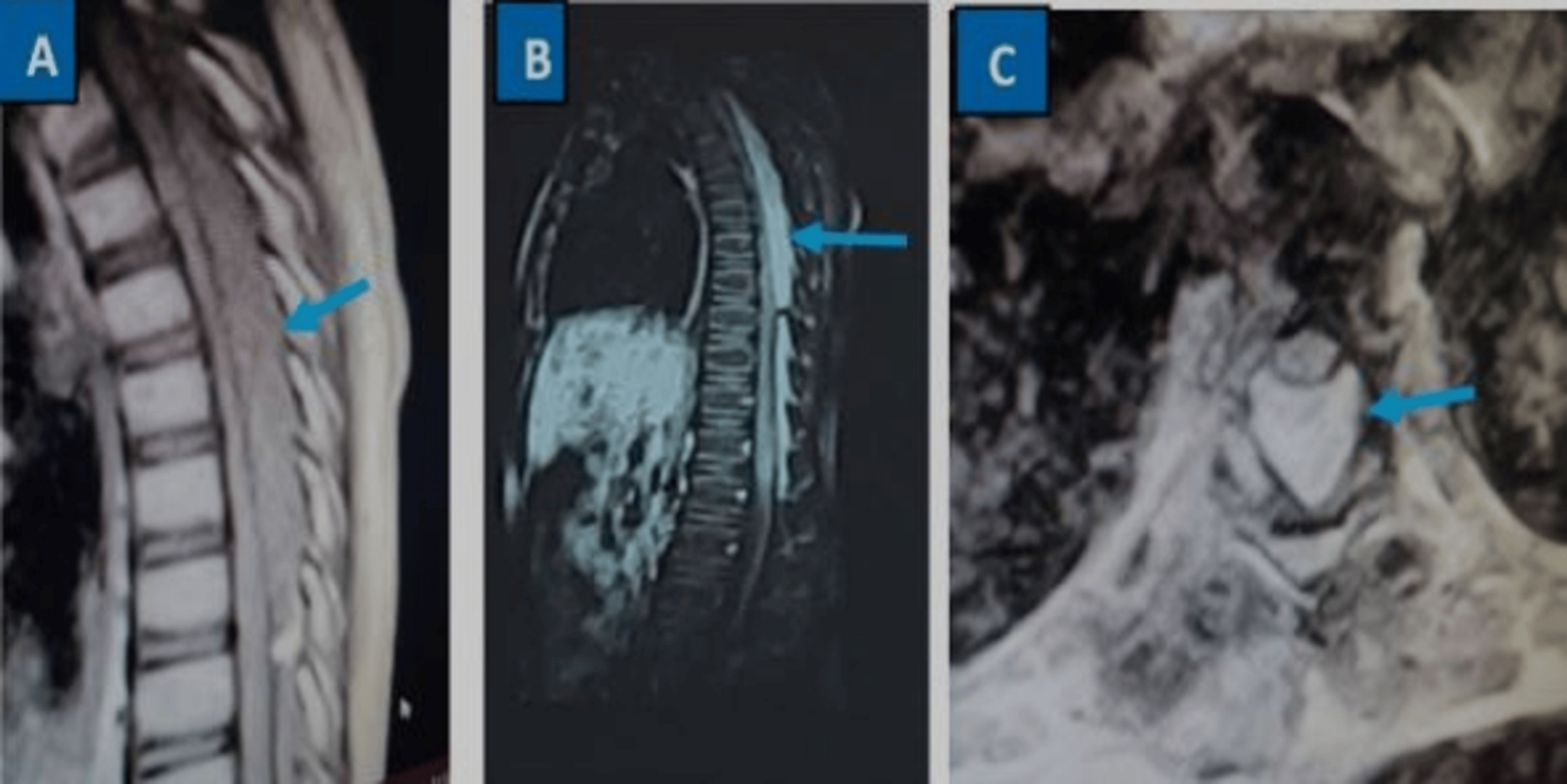
MRI of the patient's thoracolumbar spine A: Sagittal MRI (T1-weighted) shows a posterior, extra-medullary, intracanal mass extending from T1 to T10. B: Sagittal MRI (T2-weighted) displays a posterior, extra-medullary, intracanal mass extending from T1 to T10. The MRI signal characteristics suggest a compressive, fatty signal mass (blue arrow). C: Axial MRI (T2-weighted) reveals a posterior, extra-medullary, intracanal mass compressing the spinal cord (blue arrow).

Due to a lack of health insurance, the patient spent nearly 19 days in the hospital before surgery. During this time, the family gathered the necessary funds to carry out the various preoperative paraclinical examinations and the costs of the operation. A CT imaging of the thoracolumbar spine showed no vertebral or disc abnormalities. An MRI of the same spine region revealed a large, extradural, posteriorly located intracanal mass extending from T1 to T10, with signal characteristics suggesting a fat-containing lesion compressing the spinal cord (Figure 1).

On the 20th day post-admission, the patient was taken to the operating theatre for tumor excision. Under general anesthesia, the patient was positioned prone with the thorax and pelvis supported by bolsters and the head placed on a horseshoe headrest. The cervicodorsal region was cleansed with soap and running water, and the surgical site was marked with an indelible marker (from T1 to T11). Disinfection was performed using a 10% povidone-iodine dermic solution, followed by sterile draping.

**Figure 2:**
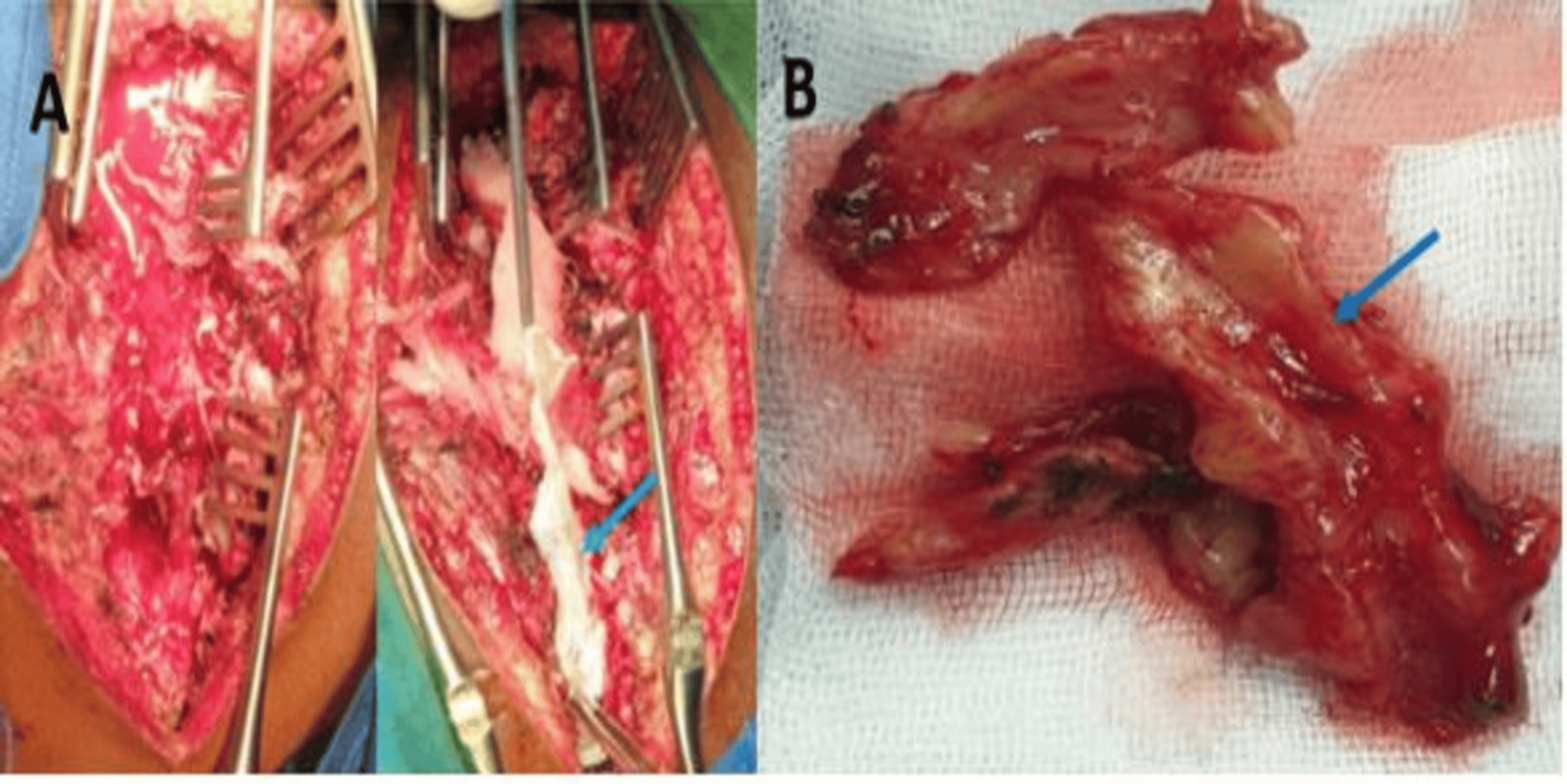
Intraoperative images A: A wide opening of the dorsal spine, with clear visualization of the extensive tumor mass (blue arrow); B: Resected tumour mass (blue arrow)

**Figure 3:**
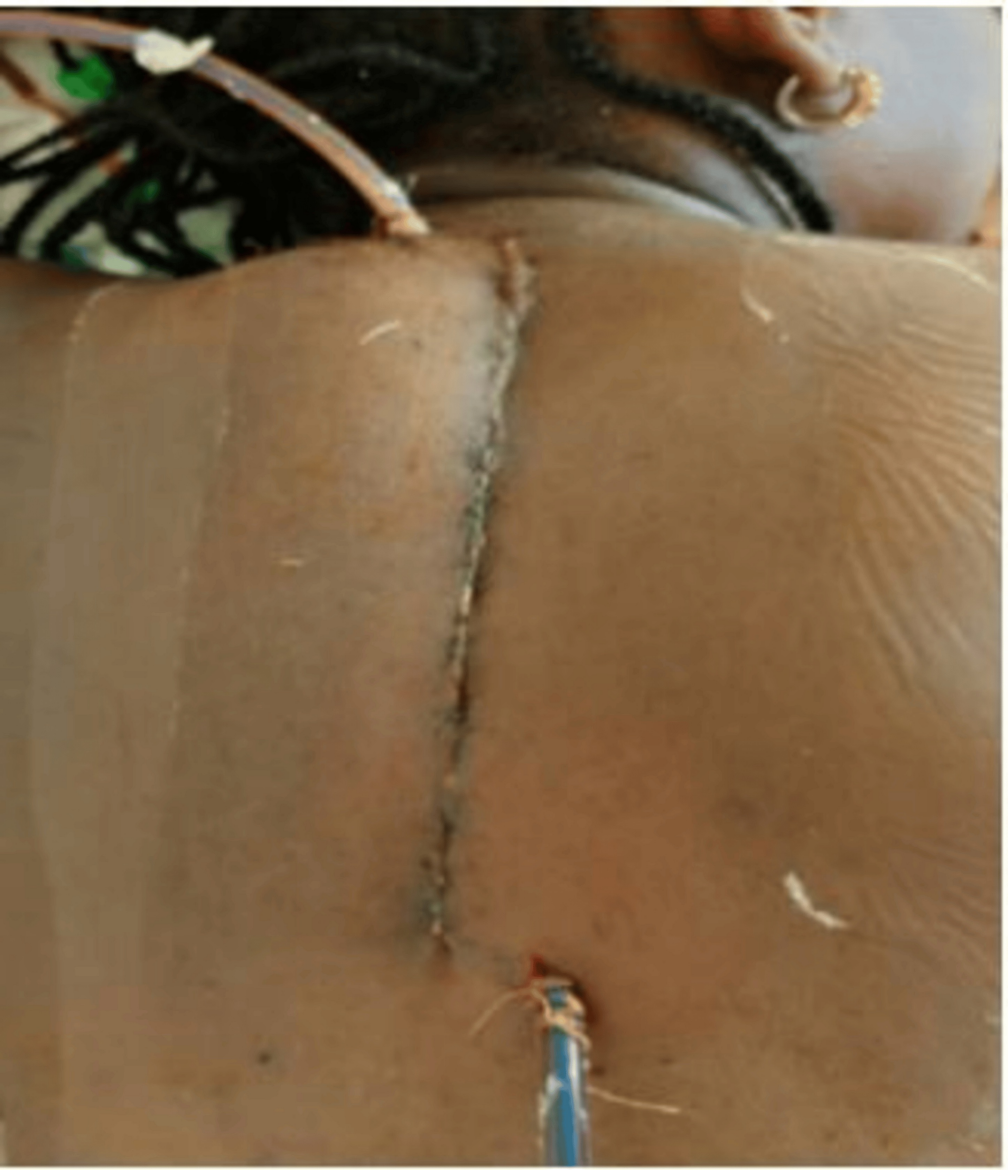
Image demonstrating dermodermal suturing for skin closure and the placement of surgical drains Image courtesy of the Neurosurgery Department.

A midline incision centered on the spinous processes from T1 to T11 was made, and paravertebral muscles were detached to expose the spinous processes and laminae. Hemostasis was achieved with a bipolar diathermy. A laminoplasty from T1 to T4, followed by a laminectomy extending to T10, was performed to access the spinal canal. A fibrofatty mass was identified intraoperatively in the posterior epidural space (Figure 2). The following surgical procedures were performed: complete excision of the mass using microsurgical instruments, repositioning of the laminar roof, layered closure of the surgical site, and placement of dual suction drains (Figure 3).

**Figure 4:**
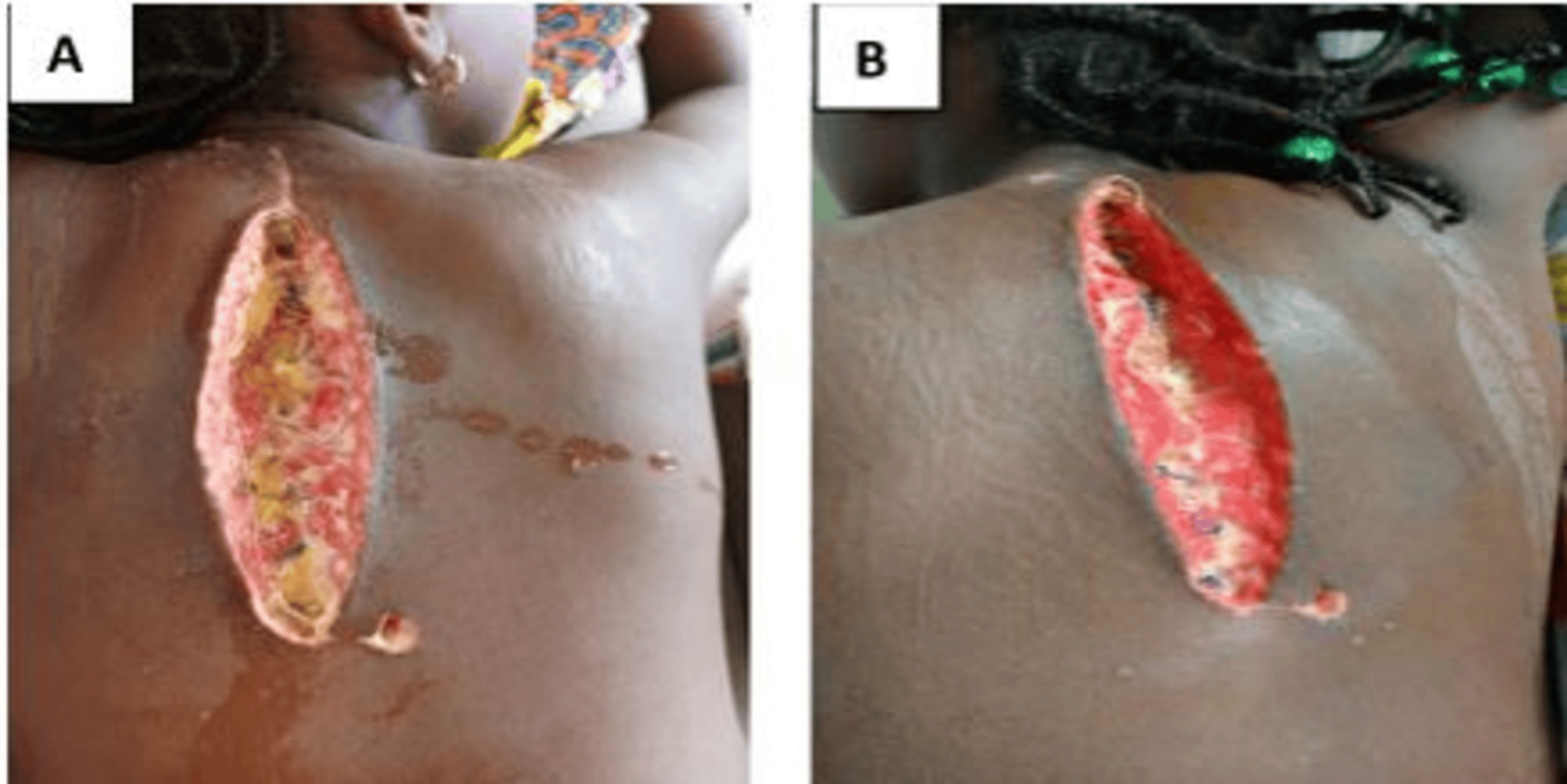
The postoperative image on day five (A) shows an infection at the surgical site, and the postoperative image taken on day 30 (B) shows the evolution of the wound.

Postoperatively, the patient received anti-inflammatory medications, analgesics, neuromodulators, and antibiotics (ceftriaxone). On day two, the patient reported sensation in the lower limbs, with initial voluntary movement and some involuntary movements. On day five, a severe surgical site infection was developed, managed with ceftriaxone and amikacin, alongside the removal of certain sutures and local wound care (Figure 4).

Histopathological findings

**Figure 5:**
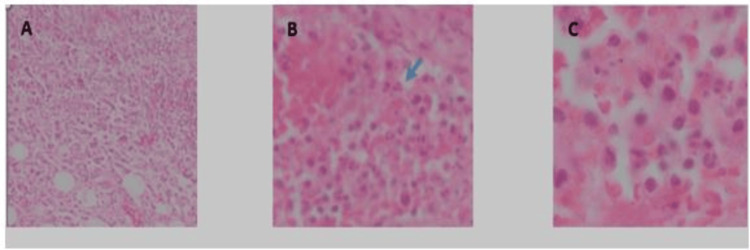
Hematoxylin and eosin stained microscopic image demonstrates fibrolipomatous inflammatory elements (blue arrows) A: Magnification 10x, B: Magnification 40x (arrow indicating a polymorphonuclear cell), C: Magnification 100x

Initial histopathological examination of the tumor specimens revealed discrepancies among the pathologists due to the heterogeneity of the histopathological elements. Findings included congestion, areas of lymphoplasmacytic infiltration, neutrophilic polymorphonuclear cells, histiocytes of varying forms, and even Langhans giant cells, fibroblasts, and adipocytes. Ultimately, a consensus was reached, diagnosing the lesion as a chronic inflammatory tumor of fibrolipomatous nature (Figure 5).

Clinical progress

**Figure 6:**
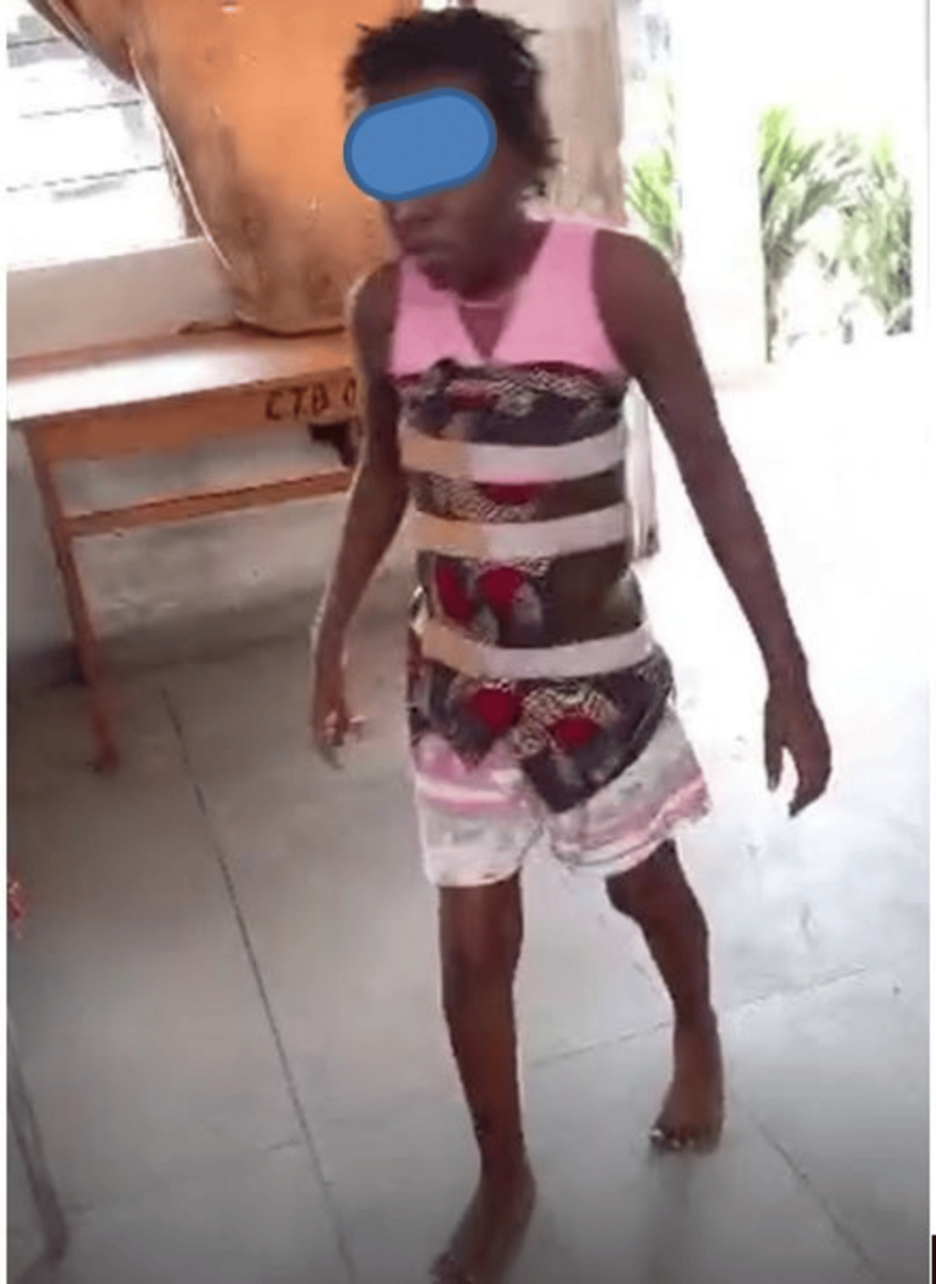
The patient standing at three months postoperatively.

On day 7, the ASIA motor score for the lower limbs remained at 20/50, with sensory function restored to 50/50. Babinski's sign was present in both lower limbs. On day 30, secondary wound closure was performed. On day 45, the onset of neurological improvement was noted. The patient continued physiotherapy at the local rehabilitation center in Kikesa. After three months of physiotherapy, neurological improvement was observed, with the ASIA motor score reaching 40/50 (Figure 6).

Following discharge from the Kikesa rehabilitation center, the patient was lost to follow-up. The contact numbers provided were no longer functional. Thus, preventing postoperative imaging from being conducted at the time of writing this manuscript.

## Discussion

Pediatric spinal tumors include a wide range of developmental tumors and other uncommon neoplasms compared to those seen in adults. Malignant processes predominate, with glial tumors, particularly astrocytomas, being the most common. Non-glial tumors are less frequent, with lymphomas and developmental tumors (e.g., epidermoid tumors, dermoid cysts, teratomas, and lipomas) being the most prevalent [3].

Inflammatory fibrolipomatous spinal masses, associated with uncontrolled proliferation of inflammatory cells and fibrous and adipose tissue, represent an extremely rare pathological entity in childhood. They are more frequently documented in adults [9-14] and infants as fibrous hamartomas [15]. To the best of our knowledge, this is the first case reported in a child. A hallmark of this inflammatory tumor is its extensive nature, often spanning multiple vertebrae. In adults, where it is more commonly described, these tumors predominantly affect the cervical and thoracic regions [14]. Histologically, these lesions have a triphasic morphology in an organoid pattern: mature adipose tissue, fibroblastic/myofibroblastic trabeculae, and small round cell nests in a myxoid matrix. However, morphologic variants have recently been described [15].

Delayed diagnosis is common in the pediatric population [7]. In our case, the diagnosis was made five months after the onset of dorsal pain. Treatment is multidisciplinary, but early and complete surgical excision remains the cornerstone for successful management. Postoperative outcomes are often better with physiotherapy. In this case, our patient regained motor function after three months of physiotherapy, highlighting another key feature of inflammatory tumors, i.e., their typically benign nature with favorable postoperative neurological outcomes. The surgical site infection observed in this case may be attributed to the lengthy incision and prolonged exposure of soft tissues during the procedure. The divergence in histopathological interpretations underscores the nature of chronic inflammatory tumors, which encompass various histopathological elements [10,11].

As previously mentioned, managing pediatric spinal tumors requires a multidisciplinary approach involving radiologists, neurosurgeons, pathologists, radiotherapists, and physiotherapists. Surgical interventions, including radical resection and, in some cases, segmental fusion with internal fixation, are recommended as first-line treatments for patients with neurological deficits or spinal instability [16]. Postoperative outcomes are generally favorable, particularly in cases that are diagnosed and treated promptly and appropriately [2].

## Conclusions

This case highlights the vast diversity of pediatric spinal tumors and their potential for favorable postoperative outcomes. It also provides evidence for the existence of benign, fibrolipomatous inflammatory tumors in children that are non-tuberculous and non-specific in origin. These tumors are notable for their ability to span multiple vertebrae and their generally benign nature.
